# Association Between Nonalcoholic Fatty Liver Disease and the Dietary Index for Gut Microbiota: A Cross‐Sectional Study

**DOI:** 10.1002/fsn3.71451

**Published:** 2026-01-12

**Authors:** Qingwan Yang, Xin Cai, Shanshan Li, Zhenghua Xiao

**Affiliations:** ^1^ The Second Clinical Medical College of Guizhou University of Traditional Chinese Medicine Guiyang China; ^2^ Department of Rheumatology and Immunology The First People's Hospital of Guiyang Guiyang China; ^3^ Department of Gastroenterology The Second Affiliated Hospital of Guizhou University of Traditional Chinese Medicine Guiyang China

**Keywords:** cross‐sectional study, dietary index, DI‐GM, gut microbiota, NAFLD, NHANES

## Abstract

Nonalcoholic fatty liver disease (NAFLD) is defined by excessive hepatic lipid deposition, which is markedly affected by dietary habits and gut microbiota. This study utilizes the Dietary Index for Gut Microbiota (DI‐GM), an established tool derived from 106 peer‐reviewed studies, to assess the effect of diet on NAFLD. By evaluating foods that modulate microbiota composition, the DI‐GM offers a robust framework for examining dietary quality and its connection to NAFLD risk in a large population. This cross‐sectional study analyzed data from the National Health and Nutrition Examination Survey (NHANES, 1999–2018) involving 101,316 participants, excluding those under 18, with incomplete data, alternative liver conditions, or excessive alcohol consumption. Finally, 5283 participants were included. NAFLD was identified using the United States Fatty Liver Index (USFLI). DI‐GM scores were calculated from two 24‐h dietary recall interviews. Multivariate logistic regression and restricted cubic splines (RCS) assessed the DI‐GM–NAFLD relationship, adjusting for demographics, lifestyle, and medical history. Subgroup and sensitivity analyses ensured robustness, while ROC curves and DeLong's test compared DI‐GM's predictive ability with Healthy Eating Index‐2015 (HEI‐2015) and Dietary Approaches to Stop Hypertension (DASH). Among 5283 participants, higher DI‐GM scores were associated with a 23.8% lower NAFLD risk (OR: 0.762, 95% CI: 0.590–0.986) in the highest versus lowest quartile. RCS analysis confirmed a linear negative association (*p* < 0.001, *p*‐nonlinearity = 0.209), which was consistent across subgroups (all *p* > 0.05). In addition, sensitivity analyses supported these findings. DI‐GM showed a higher AUC (0.867) than HEI‐2015 (0.848) and DASH (0.847; both *p* < 0.0001, DeLong's test). Elevated DI‐GM scores are inversely linked to NAFLD risk, suggesting a potential link between dietary patterns that promote beneficial gut microbiota modulation and reduced liver disease risk. This highlights the potential of DI‐GM‐guided dietary interventions for NAFLD prevention. Nevertheless, additional studies are required to clarify the underlying mechanisms.

## Graphical abstract

Higher Dietary Index for Gut Microbiota (DI‐GM) scores, derived from NHANES dietary recalls, were associated with a 23.8% lower Nonalcoholic fatty liver disease (NAFLD) risk (OR: 0.762, 95% CI: 0590–0.986), with a linear relationship (*p*‐nonlinearity = 0.209). DI‐GM outperformed HEI‐2015 and DASH in predicting NAFLD (AUC: 0.867 vs. 0.848, 0.847; *p* < 0.0001). These findings highlight dietary modulation of gut microbiota as a protective factor against NAFLD.

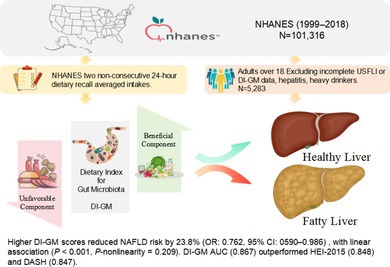

**Abbreviations**
CI: confidence intervals
DI‐GM: Dietary Index for Gut Microbiota
NAFLD: nonalcoholic fatty liver disease
NHANES: National Health and Nutrition Examination Survey
OR: odds ratio
PA‐MET: physical activity was expressed as metabolic equivalent task
PIR: poverty‐to‐income ratio
RCS: restricted cubic spline
USFLI: United States Fatty Liver Index

## 1 Introduction

Nonalcoholic fatty liver disease (NAFLD) is defined by fat deposition in liver cells in the absence of substantial alcohol intake or other discernible etiologies (Chalasani et al. 2018), typically confirmed via liver biopsy (Singh et al. 2015). It is a major liver disorder contributing to global disease burden, with a prevalence estimated between 25.24% and 33.90% (Li et al. 2019; Younossi et al. 2016). Moreover, projections indicate that the prevalence of NAFLD will rise to nearly 56% within the next decade (Huang et al. 2021). The condition often progresses in a subclinical manner due to its asymptomatic nature, posing significant health risks on hepatocyte inflammation (Pouwels et al. 2022), fibrosis and cirrhosis (Younossi 2019). Additionally, NAFLD is associated with heightened risks of depression (Li, Li, et al. 2024), type 2 diabetes mellitus (T2DM) (Lindfors et al. 2025), coronary heart disease (CHD) (Gries et al. 2025), and cancers (Ahmadishoar et al. 2025; Park et al. 2024), highlighting its importance as a global health issue with extensive clinical (Younossi et al. 2023) and economic consequences.

The gut microbiota is vital for multiple physiological processes, including lipid absorption, metabolic processing, detoxification, and endocrine regulation (Liu et al. 2025; Wu et al. 2023). Recent studies have underscored its impact on crucial risk factors of NAFLD, including dyslipidemia and T2DM (Nychas et al. 2025). Specifically, the imbalance of gut microbiota is distinguished by a heightened Firmicutes/Bacteroidetes ratio and Proteobacteria proliferation, which may increase intestinal permeability, lead to the migration of endotoxins, and trigger inflammation across multiple organs, including the liver (Jasirwan et al. 2021; Li, Cai, et al. 2024).

Dietary composition plays a crucial role in health maintenance, with suboptimal patterns intricately linked to NAFLD through disruptions in gut microbiota dynamics and metabolic outputs (Mims et al. 2021). Real‐world evidence highlights how such patterns influence NAFLD progression. For instance, Western diets promote hepatic lipid synthesis and cholesterol absorption by altering microbiota (Wang et al. 2022), while poor habits trigger liver inflammation via immune dysregulation and pathway activation (Yang et al. 2023). In contrast, nutrient‐rich patterns like the Mediterranean diet foster a healthier microbial environment, thereby mitigating dysbiosis, reducing inflammation, and offering therapeutic benefits (Rahimlou et al. 2022; Xiong et al. 2024).

Nonetheless, the overall impact of intricate dietary components on health remains incompletely understood. The Dietary Index for Gut Microbiota (DI‐GM), developed from 106 peer‐reviewed studies (Kase et al. 2024), pinpoints 14 foods or nutrients influencing gut microbiota and categorizes them as advantageous or harmful. These include beneficial components such as avocados, broccoli, whole grains, cranberries, coffee, green tea, soybean, and fermented foods (e.g., cheese, kefir). Also, harmful components include red meat, processed meats, refined grains, and high‐fat diets (> 40% energy). Additionally, they further developed and validated the DI‐GM via the NHANES cohort (Kase et al. 2024). Unlike general dietary quality indices like the Healthy Eating Index‐2015 (HEI‐2015) or Dietary Approaches to Stop Hypertension (DASH), DI‐GM specifically targets gut microbiota and microbiota‐mediated pathways, such as SCFA production and bile acid metabolism, which are implicated in NAFLD pathogenesis.

Given the accessibility, cost‐effectiveness, and greater adherence of dietary interventions, elucidating the linkage between DI‐GM and NAFLD could be imperative. By leveraging NHANES 1999–2018 data—characterized by its nationally representative cohort, rigorous dietary recall methodology, and extensive sociodemographic and clinical covariates—this study aims to elucidate the intricate relationship between DI‐GM and NAFLD. We hypothesize that higher DI‐GM scores, reflecting greater consumption of microbiota‐beneficial foods, are inversely associated with NAFLD risk. Additionally, the association is potentially mediated by enhanced SCFA production and improved bile acid signaling, offering novel insights for NAFLD prevention.

## 2 Methods

### 2.1 Study Design

**FIGURE 1:**
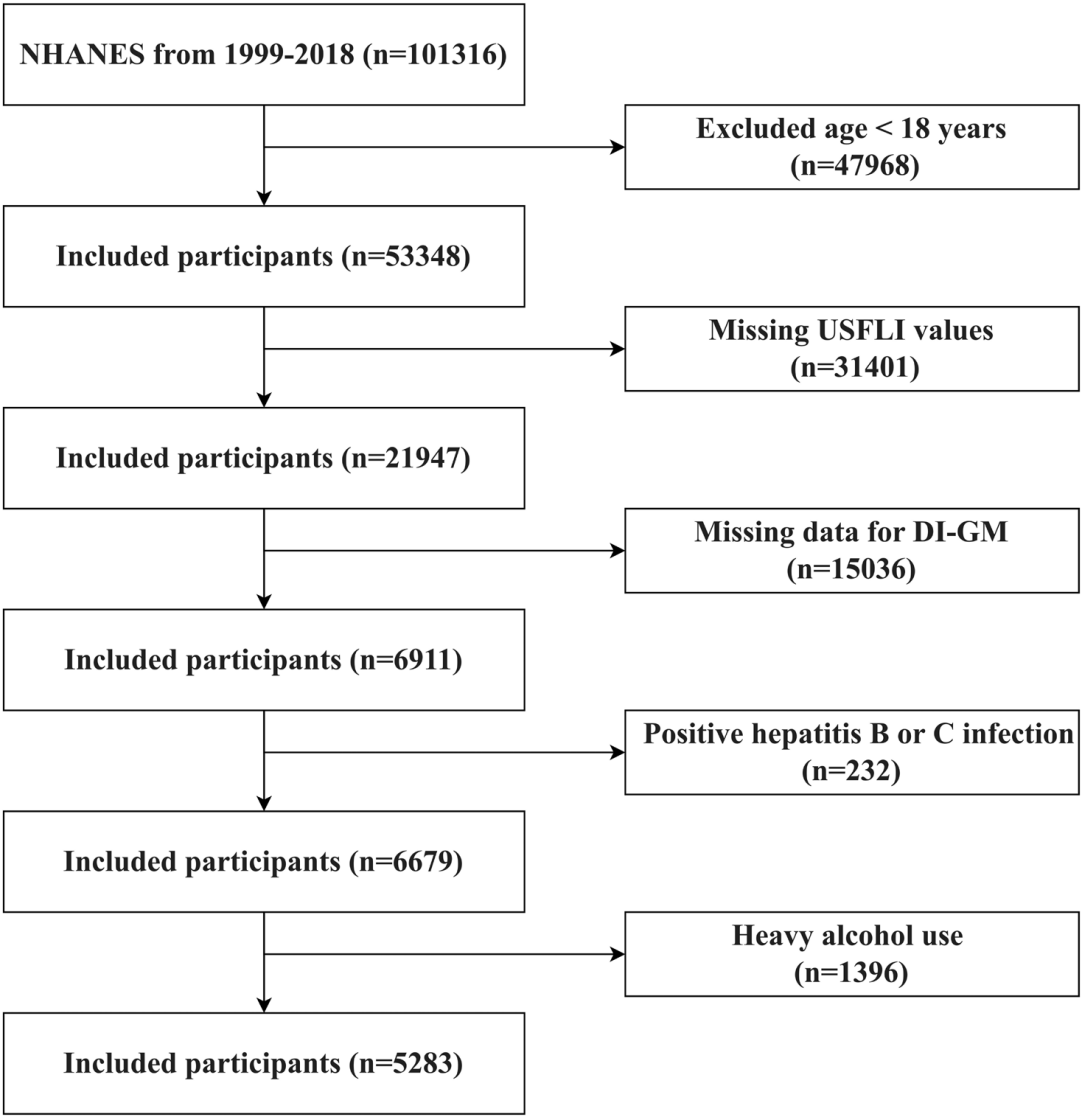
Flowchart of participant selection.

Data of our study were sourced from the NHANES, which represents a complex, multistage and probability‐based survey, aiming at assessing the nutritional and health status among U.S. population. Individuals aged over 18 years were included with written informed consent provided (Yang et al. 2023). The exclusion criteria were: (1) those lacking complete US Fatty Liver Index (USFLI) data (*n* = 31,401); (2) those without full DI‐GM data (*n* = 15,036); (3) those testing positive for hepatitis B or C (*n* = 232); and (4) those who consume > 3 alcoholic beverages per day for men and > 2 for women (*n* = 1396). Figure 1 depicts the process of the enrollment of participants.

### 2.2 Dietary Intake Assessment

Dietary data were derived from two non‐consecutive 24‐h dietary recalls provided by NHANES. The initial recall was carried out in person, while the subsequent one was completed via telephone between 3 and 10 days later. Average nutrient intake across these 2 days was calculated utilizing the Food and Nutrient Database for Dietary Studies. While 24‐h recalls are subject to recall bias, averaging data from two non‐consecutive days mitigates this by providing a more representative estimate of usual intake and reducing random error associated with single‐day reporting.

### 2.3 Construction and Scoring of Three Dietary Indices

The 14 dietary components that make up the DI‐GM were determined using the grading system devised by Kase et al. (2024). These components, identified through a systematic analysis of 106 studies, are grouped as beneficial or harmful according to their influence on gut microbiota. If consumption was greater than or equal to the sex‐specific median, items linked to a beneficial gut microbiota received 1 score; otherwise the score would be 0. For gut microbiota‐harming foods, 0 was given if consumption was at or above the sex‐specific median or 40% (high‐fat diets); otherwise, 1 score was issued. Sex‐specific medians, derived internally from the study population rather than external references, were utilized to account for inherent physiological differences in dietary intake and energy requirements. Additionally, a 40% threshold was applied to define high‐fat diets, consistent with established standards. Information of these components and scoring methods are provided in Table S1 (Kase et al. 2024).

To further examine the utility of the DI‐GM, we additionally calculated the Healthy Eating Index (HEI)‐2015 and Dietary Approaches to Stop Hypertension (DASH) scores. The HEI‐2015 encompasses 13 dietary components (Krebs‐Smith et al. 2018), which could be classified as adequacy and moderation ones. Specifically, within the adequacy components, Total Fruits, Whole Fruits, Total Vegetables, Greens and Beans, Total Protein Foods, Seafood and Plant Proteins receive a maximum of 5 points, respectively. Whole Grains, Dairy, and Fatty Acids (within the adequacy components), as well as all moderation components (Refined Grains, Sodium, Added Sugars, and Saturated Fats), each receive a maximum of 10 points. For all components except Fatty Acids, scores are determined by nutrient density per kilocalorie (energy percentage). In brief, the HEI‐2015 total score ranges from 0 to 100, with higher scores suggesting superior diet quality.

Similar to the HEI‐2015, the DASH score is based on eight food components (Hekmatdoost et al. 2016). Fruits, vegetables, whole grains, low‐fat dairy products, and legumes/nuts attain 5 points at recommended intakes, decreasing proportionally to a minimum of 1 point for reduced intakes. In contrast, saturated fats, sodium, and added sugars attain 5 points at minimal intakes, decreasing to 1 point at maximal intakes.

### 2.4 Definition of NAFLD

The USFLI is a noninvasive indicator for liver diseases included in NHANES from 1988 to 1994, which incorporates factors such as ethnicity, age, insulin levels, blood glucose, waist circumference, and gamma‐glutamyl transferase (GGT) activity. For this study, a USFLI score over 30 was adopted as the diagnostic criterion for NAFLD as suggested by Ruhl and Everhart, with an area under the receiver operating characteristic curve of 0.8 (sensitivity: 62%; specificity: 88%) (Ruhl and Everhart 2015). However, this approach may introduce misclassification bias, potentially underestimating NAFLD prevalence in cases with lower steatosis levels. Individuals with hepatitis B or C, other hepatic diseases, or overconsumption of alcohol were ruled out before applying this criterion.

### 2.5 Covariates

This study assessed various demographic features including gender, age, ethnicity, education level, marital status, and smoking status (former, never, or current smoker) to ascertain potential confounders. The poverty‐to‐income ratio (PIR) was divided into three tiers: low (PIR < 1.3), medium (1.3 < PIR < 3.5), and high (PIR > 3.5). BMI was segmented into underweight (< 25), overweight (25–30), and obese (≥ 30). Physical activity (PA) was expressed as metabolic equivalent task (MET) minutes per week. Data on PA were obtained through standardized NHANES questionnaires. Total PA‐MET (min/week) was computed based on the 2011 Compendium of Physical Activities and NHANES‐specific protocols, integrating intensity (MET values), frequency, and duration for each reported activity (Wei et al. 2024). In addition, medical histories were also reviewed, focusing on hyperlipidemia, diabetes, and hypertension. Moreover, diagnosis of these diseases adhered to guidelines of America of that time (Carey and Whelton 2018; Elsayed et al. 2023; National Cholesterol Education Program (US) 2002).

### 2.6 Statistical Analysis

All analyses were performed with R software (version 4.3.1). Survey weights were not applied, as the study population was selectively derived from NHANES after applying strict inclusion/exclusion criteria, prioritizing internal validity and associations within this subsample over national representativeness. This approach is appropriate for exploratory cross‐sectional analyses focused on hypothesis testing rather than prevalence estimation, though it limits generalizability to the broader U.S. population. Normally distributed continuous variables were reported as means ± standard deviations (SD) and compared using *t*‐tests; skewed variables were shown as medians with interquartile ranges (P25–P75) and compared using Wilcoxon tests. Categorical variables were presented as frequencies (percentages) and compared using Chi‐squared tests. Multicollinearity among covariates was assessed using variance inflation factors (VIFs); values exceeding 5 indicate collinearity (Vatcheva et al. 2016). Specifically, the DI‐GM scores of participants were classified into quartiles: Q1 (0–3), Q2 (4), Q3 (5), and Q4 (6–14). Additionally, multivariable logistic regression was conducted, employing odds ratios (ORs) and 95% confidence intervals (CIs) to measure associated risks. The analysis included three progressively comprehensive models: Model 1 (unadjusted), Model 2 (adjusted for age, gender, ethnicity, educational level, marital status, and PIR), and Model 3 (further adjusted for PA‐MET, smoking status, BMI, hypertension, diabetes, and hyperlipidemia), with *p* for trends across quartiles evaluated. Nonlinear correlations between DI‐GM and NAFLD were examined using restricted cubic spline (RCS) in Model 3, with four knots specified at the 5th, 35th, 65th, and 95th percentiles of DI‐GM scores to ensure flexibility and reproducibility. Subgroup analyses stratified by covariates evaluated heterogeneity, with interaction tests utilized to identify effect modifiers. Furthermore, sensitivity analyses were performed to assess robustness: (1) multiple imputation by chained equations (MICE) to generate five datasets, with pooled estimates using Rubin's rules; (2) complete‐case analysis. To assess the utility of the DI‐GM in comparison with the HEI‐2015 and DASH score, receiver operating characteristic (ROC) curves were developed to assess their predictive capability for NAFLD, with area under the curve (AUC) values serving as the principal measure. DeLong's test was also employed to ensure the strength of DI‐GM. All statistical significance established at *p* < 0.05 (two‐tailed).

## 3 Results

### 3.1 Baseline Characteristics

From the initial pool of 101,316 participants, a final cohort of 5283 individuals was established and stratified into quartiles according to DI‐GM. Participants with higher DI‐GM scores (Q4) showed a higher proportion of females, greater levels of educational attainment, increased PIR and BMI, lower incidence of hypertension, diabetes, and hyperlipidemia compared to individuals in Q1. Detailed baseline characteristics of these participants are delineated in Table 1.

**TABLE 1:** Basic characteristics of the participants by DI‐GM scores.

DI_GM quartile	Q1 (0–3)	Q2 (4)	Q3 (5)	Q4 (6–11)	*p*
*N*	1137	1262	1239	1645	
Age (years), mean (SD)	48.00 (18.26)	47.56 (18.44)	49.51 (18.27)	52.19 (17.46)	< 0.001
PA‐MET (min/week), mean (SD)	5085.46 (7142.53)	4226.87 (5768.08)	3784.95 (5655.81)	3779.92 (6289.84)	< 0.001
Gender (%)					< 0.00 1
Male	594 (52.24)	569 (45.09)	577 (46.57)	718 (43.65)	
Female	543 (47.76)	693 (54.91)	662 (53.43)	927 (56.35)	
Ethnicity (%)					< 0.001
Mexican American	160 (14.07)	179 (14.18)	169 (13.64)	178 (10.82)	
Other Hispanic	124 (10.91)	142 (11.25)	116 (9.36)	148 (9.00)	
Non‐Hispanic White	476 (41.86)	541 (42.87)	577 (46.57)	911 (55.38)	
Non‐Hispanic Black	289 (25.42)	282 (22.35)	250 (20.18)	211 (12.83)	
Other Race	88 (7.74)	118 (9.35)	127 (10.25)	197 (11.98)	
Educational level (%)					< 0.001
High school or less	569 (50.04)	539 (42.71)	473 (38.18)	507 (30.82)	
Some college	307 (27.00)	367 (29.08)	368 (29.70)	478 (29.06)	
College graduate or higher	221 (19.44)	312 (24.72)	361 (29.14)	635 (38.60)	
Not recorded	40 (3.52)	44 (3.49)	37 (2.99)	25 (1.52)	
Marital status (%)					< 0.001
Married	589 (51.80)	613 (48.57)	685 (55.29)	984 (59.82)	
Divorced or separated	210 (18.47)	267 (21.16)	249 (20.10)	326 (19.82)	
Never married	202 (17.77)	249 (19.73)	198 (15.98)	221 (13.43)	
Not recorded	136 (11.96)	133 (10.54)	107 (8.64)	114 (6.93)	
Poverty to income ratio (%)					< 0.001
< 1.3	331 (29.11)	367 (29.08)	300 (24.21)	300 (18.24)	
1.3–3.5	421 (37.03)	431 (34.15)	417 (33.66)	503 (30.58)	
> 3.5	297 (26.12)	358 (28.37)	416 (33.58)	725 (44.07)	
Not recorded	88 (7.74)	106 (8.40)	106 (8.56)	117 (7.11)	
Smoking status (%)					< 0.001
Former	678 (59.63)	798 (63.23)	781 (63.03)	1048 (63.71)	
Never	195 (17.15)	170 (13.47)	165 (13.32)	165 (10.03)	
Current	250 (21.99)	277 (21.95)	279 (22.52)	416 (25.29)	
Not recorded	14 (1.23)	17 (1.35)	14 (1.13)	16 (0.97)	
Body mass index (%)					< 0.001
< 25	296 (26.03)	407 (32.25)	387 (31.23)	592 (35.99)	
25–29.9	375 (32.98)	405 (32.09)	405 (32.69)	592 (35.99)	
≥ 30	465 (40.90)	447 (35.42)	443 (35.75)	457 (27.78)	
Not recorded	1 (0.09)	3 (0.24)	4 (0.32)	4 (0.24)	
Hypertension (%)					0.153
No	643 (56.55)	747 (59.19)	755 (60.94)	987 (60.00)	
Yes	494 (43.45)	515 (40.81)	484 (39.06)	658 (40.00)	
Diabetes (%)					0.002
No	867 (76.25)	1006 (79.71)	1007 (81.28)	1362 (82.80)	
Yes	259 (22.78)	241 (19.10)	222 (17.92)	268 (16.29)	
Not recorded	11 (0.97)	15 (1.19)	10 (0.81)	15 (0.91)	
Hyperlipidemia (%)					0.677
No	311 (27.35)	371 (29.40)	342 (27.60)	461 (28.02)	
Yes	826 (72.65)	891 (70.60)	897 (72.40)	1184 (71.98)	

### 3.2 Association Between the DI‐GM and NAFLD

**FIGURE 2:**
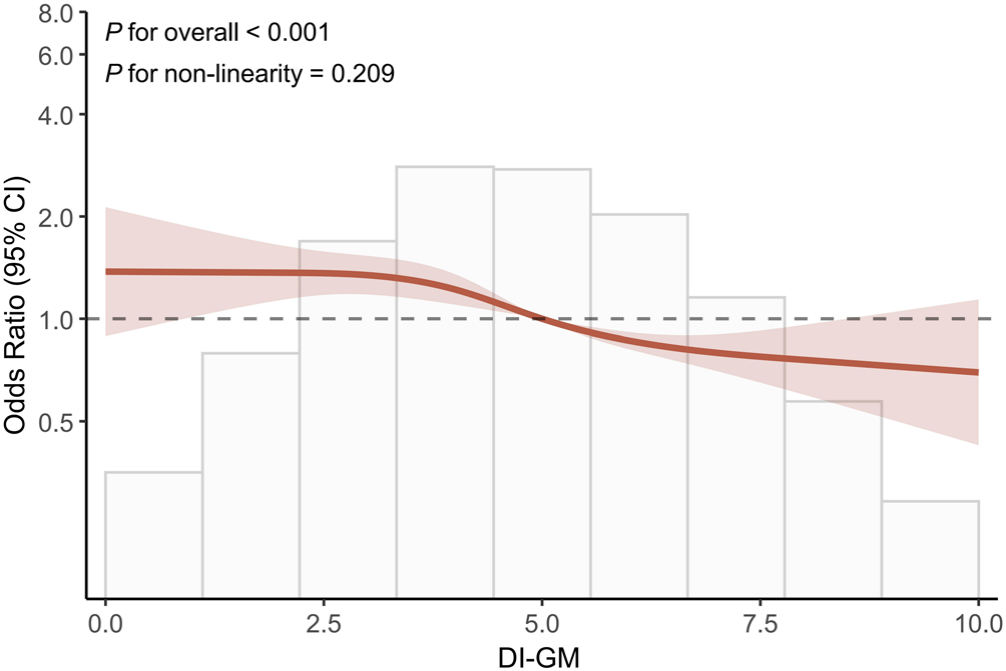
RCS analysis of the association between DI‐GM scores and NAFLD risk, adjusted for all covariates in Model 3 (age, gender, ethnicity, educational level, marital status, PIR, PA‐MET, smoking status, BMI, hypertension, diabetes, and hyperlipidemia). The solid line represents the OR, with dashed lines indicating 95% confidence intervals. The analysis reveals a linear dose–response relationship, with progressively decreasing NAFLD risk as DI‐GM scores increase (*p* < 0.001; *p*‐nonlinearity = 0.209), without evidence of threshold effects. Knots were placed at the 5th, 35th, 65th, and 95th percentiles of DI‐GM scores.

Multivariable logistic regression analyses revealed an inverse correlation between DI‐GM and the prevalence of NAFLD. Specifically, the OR was 0.913 (95% CI: 0.881–0.946; *p* < 0.001) in Model 1, 0.891 (95% CI: 0.857–0.927; *p* < 0.001) in Model 2, and 0.943 (95% CI: 0.891–0.998; *p* = 0.044) in Model 3 (Table 2). In Model 1, participants in Q3 (OR: 0.820, 95% CI: 0.692–0.972) and Q4 (OR: 0.642, 95% CI: 0.545–0.755) displayed notably lower risks of NAFLD compared to those in Q1, evidencing a pronounced trend (*p* for trend < 0.001). Subsequent adjustments in Model 2 confirmed the persistence of this trend, with decreased risks in Q3 (OR: 0.796, 95% CI: 0.664–0.954) and Q4 (OR: 0.592, 95% CI: 0.496–0.707). Further comprehensive adjustments in Model 3 maintained the significant reduction in NAFLD risk in Q4 relative to Q1 (OR: 0.762, 95% CI: 0.590–0.986), substantiated by a statistically significant trend (*p* for trend = 0.039). Additionally, we conducted RCS analysis, adjusting for all the covariates in Model 3, to assess potential nonlinear relationships. The results from RCS analysis revealed a linear dose–response correlation between higher DI‐GM scores and a reduced risk of NAFLD (*p* < 0.001, *p*‐nonlinearity = 0.209). This suggested that as DI‐GM scores increase, the risk of NAFLD decreases progressively, without the presence of threshold effects (Figure 2).

**TABLE 2:** Association between DI‐GM and NAFLD by multivariable logistic regression.

Exposure	Model 1	Model 2	Model 3
	OR (95% CI)	OR (95% CI)	OR (95% CI)
	*p*	*p*	*p*
DI_GM	0.913 (0.881, 0.946) < 0.001	0.891 (0.857, 0.927) < 0.001	0.943 (0.891, 0.998) 0.044
DI_GM quartile			
Q1	1	1	1
Q2	0.856 (0.723, 1.013) 0.070	0.881 (0.736, 1.054) 0.165	0.992 (0.762, 1.291) 0.953
Q3	0.820 (0.692, 0.972) 0.022	0.796 (0.664, 0.954) 0.014	1.004 (0.771, 1.309) 0.973
Q4	0.642 (0.545, 0.755) < 0.001	0.592 (0.496, 0.707) < 0.001	0.762 (0.590, 0.986) 0.039
*p* for trend	< 0.001	< 0.001	0.039

*Note:* Model 1: Non‐adjusted. Model 2: Adjusted for age, gender, ethnicity, educational level, marital status, poverty to income ratio. Model 3: Adjusted for age, physical activity MET, gender, ethnicity, educational level, marital status, poverty to income ratio, smoking status, body mass index, hypertension, diabetes, hyperlipidemia.

### 3.3 Subgroup Analysis

Participants were stratified based on covariates, with results shown in Table 3. A negative association between the DI‐GM and NAFLD risk was observed among participants aged ≤ 39 or 40–59 years, male and female, non‐hypertensive individuals, non‐diabetic individuals, non‐hyperlipidemic and hyperlipidemic patients (all *p* < 0.05). Furthermore, no significant interaction effects were identified across these subgroups (all *p* > 0.05). These findings indicated the stable association across diverse populations, without significant variation in magnitude or direction in any subgroup. To evaluate potential multicollinearity among covariates in the multivariable logistic regression model (e.g., between BMI and hyperlipidemia), we calculated the VIF for each covariate (Table 4). All VIF values ranged from 1.1 to 1.7, substantially below the conventional threshold of 5. These results indicated no significant multicollinearity, thereby supporting the reliability of the regression parameter estimates.

**TABLE 3:** Subgroup analysis of the association between DI‐GM and NAFLD.

Subgroups	OR (95% CI)	*p* for interaction
	*p*	
Age		0.604
≤ 39	0.90 (0.79, 1.02) 0.009	
40–59	0.87 (0.79, 0.97) 0.011	
≥ 60	0.97 (088, 1.06) 0.450	
Gender		0.439
Male	0.93 (0.87, 0.99) 0.031	
Female	0.90 (0.83, 0.97) 0.006	
Ethnicity		0.835
Mexican American	0.860 (0.720, 1.010) 0.073	
Other Hispanic	0.880 (0.730, 1.070) 0.216	
Non‐Hispanic White	0.970 (0.900, 1.050) 0.487	
Non‐Hispanic Black	0.860 (0.690, 1.070) 0.184	
Body mass index		0261
< 25	0.920 (0.750, 1.150) 0.476	
25–29.9	0.930 (0.850, 1.010) 0.103	
≥ 30	0.960 (0.880, 1.040) 0.280	
Smoking status		0.457
Never	0.94 (0.880, 1.020) 0.124	
Current	0.960 (0.810, 1.150) 0.658	
Former	0.920 (0.820, 1.030) 0.157	
Diabetes		0.270
No	0.930 (0.870, 0.909) 0.021	
Yes	0.960 (0.850, 1.080) 0.452	
Hypertension		0.093
No	0.880 (0.810, 0.960) 0.004	
Yes	0.970 (0.900, 1.050) 0.482	
Hyperlipidemia		0.539
No	0.860 (0.750, 0.980) 0.026	
Yes	0.910 (0.860, 0.960) < 0.001	

**TABLE 4:** Variance inflation factor (VIF) results for covariates.

Covariate	VIF value
Age (years)	1.7
PA‐MET (min/week)	1.1
Gender (%)	1.1
Ethnicity (%)	1.1
Educational level (%)	1.4
Marital status (%)	1.3
Poverty to income ratio (%)	1.3
Smoking status (%)	1.1
Body mass index (%)	1.2
Hypertension (%)	1.4
Diabetes (%)	1.2
Hyperlipidemia (%)	1.2

### 3.4 Sensitivity Analyses

Two sensitivity analyses were conducted to ensure robustness, and no major differences were observed compared to the primary analyses. Analysis restricted to complete cases (*n* = 4793) produced similar results (Model 3 OR: 0.926, 95% CI: 0.873–0.982, *p* = 0.010, Table S2), suggesting the insignificant impact of missing data on our findings. Multiple imputation created five complete datasets, with pooled analyses yielding consistent results (Model 3 OR: 0.930, 95% CI: 0.877–0.986, *p* = 0.015, Table S3), which indicated no statistical inferences by imputation. These sensitivity analyses collectively strengthened our primary findings, confirming a robust inverse association between DI‐GM and NAFLD risk.

### 3.5 Comparison of DI‐GM, HEI‐2015 and DASH Performance Using ROC

**FIGURE 3:**
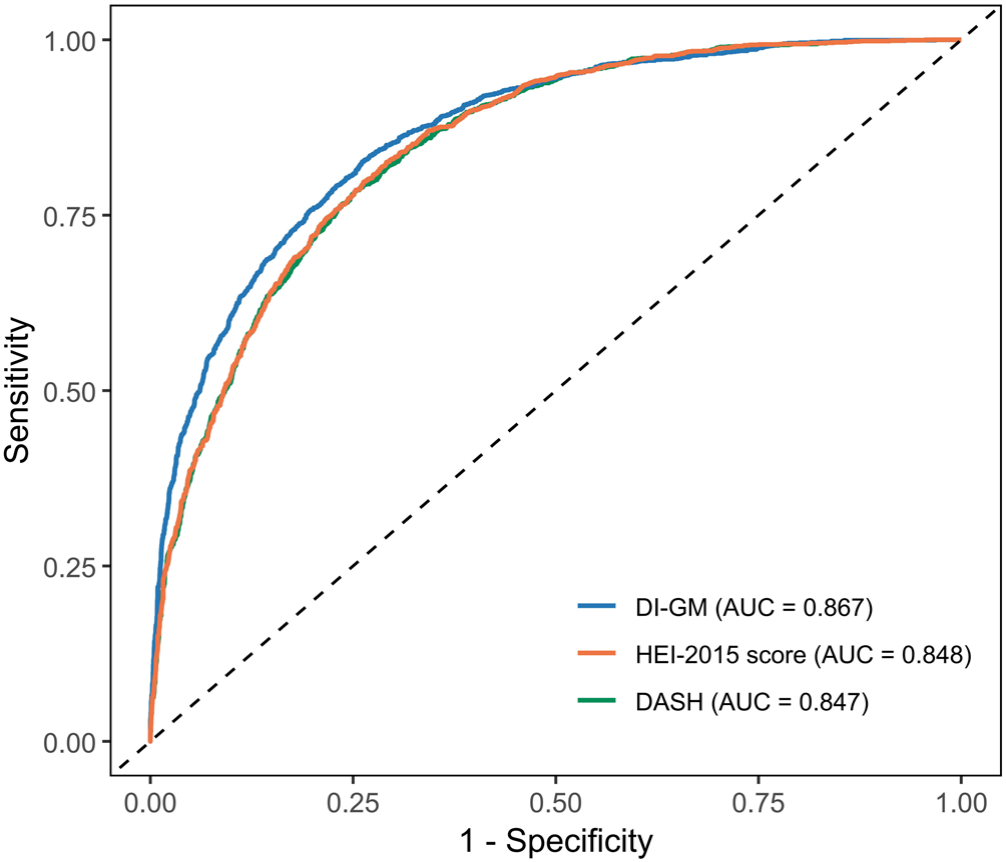
ROC curves comparing the predictive performance of DI‐GM, HEI‐2015, and DASH scores for NAFLD risk. The area under the curve (AUC) values are 0.867 (95% CI: 0.857–0.877) for DI‐GM, 0.848 (95% CI 0.838–0.859) for HEI‐2015, and 0.847 (95% CI: 0.836–0.857) for DASH, with DI‐GM showing significantly superior discrimination (DeLong's test *p* < 0.0001 vs. both).

ROC curve analysis (Figure 3; Table 5) showed that DI‐GM performed better in predicting NAFLD risk than HEI‐2015 and DASH. Specifically, the AUC values were 0.867 (95% CI: 0.857–0.877; *p* < 0.001) for DI‐GM, 0.848 (95% CI: 0.838–0.859; *p* < 0.001) for HEI‐2015, and 0.847 (95% CI: 0.836–0.857; *p* < 0.001) for DASH. DeLong's test indicated significant differences for DI‐GM versus both HEI‐2015 and DASH (both *p* < 0.0001). For sensitivity, the DI‐GM (0.825) was comparable to the HEI‐2015 (0.826) but outperformed the DASH (0.786). DI‐GM also demonstrated higher accuracy (0.772) than HEI‐2015 (0.746) and DASH (0.758). Furthermore, DI‐GM outperformed both indices in positive predictive value (PPV) and negative predictive value (NPV) (Table 5).

**TABLE 5:** Diagnostic performance metrics for DI‐GM, HEI‐2015, and DASH scores in predicting NAFLD risk at optimal Youden Index thresholds.

	Youden index	Thscoreold	Sensitivity	Specificity	Accuracy	PPV	NPV
DI‐GM	0.577	0.292	0.825	0.752	0.772	0.602	0.899
HEI‐2015	0.535	0.258	0.826	0.709	0.746	0.565	0.899
DASH	0.532	0.288	0.786	0.746	0.758	0.587	0.884

Abbreviations: NPV, negative predictive value; PPV, positive predictive value.

## 4 Discussion

Our analysis revealed a significant inverse linear dose–response relationship between DI‐GM scores and NAFLD incidence (OR = 0.943, 95% CI: 0.891–0.998) after adjusting for demographics, lifestyle factors, and comorbidities. Higher DI‐GM scores, reflecting greater intake of microbiota‐beneficial foods such as avocados, whole grains, and fermented products, correlate with progressively lower NAFLD risk. As a consequence, clinicians can use DI‐GM scores to inform personalized dietary recommendations, prioritizing these foods to reduce NAFLD risk in high‐risk populations. The association was more pronounced in hyperlipidemic subgroups, likely due to the influence of these foods on lipid metabolism and gut barrier function. Conversely, the association was less consistent in hypertensive subgroups, possibly due to sodium intake confounding effects, necessitating further research.

Globally, NAFLD stands as the predominant chronic liver condition with the highest prevalence of 33.9% (Younossi et al. 2016), which is 42.6% in the Middle East‐North Africa region (Henry et al. 2022). In addition to hepatic fat accumulation, NAFLD progression can lead to severe complications, including hepatitis and liver fibrosis. NAFLD constitutes a significant public health issue owing to high incidence and possible comorbidities, especially in economically developed regions (Henry et al. 2022). Its advancement and progression are notably influenced by unhealthy dietary habits and disruptions in gut microbiota.

NAFLD reflects abnormal fat distribution in the body, primarily due to excessive fat intake, synthesis and redistribution (lean NAFLD) (Hsu et al. 2024; Kim et al. 2016). Unhealthy dietary habits represent the first critical cause for NAFLD, characterized by energy intake surpassing physiological needs and insufficient consumption of beneficial components that support metabolism (Juanola et al. 2021). The excessive intake of red and processed meats, refined grains, and high‐fat diets significantly induces metabolic stress and increases health risks, despite their widespread consumption (Charlot et al. 2024; Hadefi et al. 2023). A study involving 77,795 women without NAFLD indicated that elevated red meat consumption was correlated with a higher incidence of NAFLD. Moreover, women who consumed two or more servings of red meat every day showed higher NAFLD (HR: 1.52, 95% CI: 1.23, 1.89) (Kim et al. 2022). The presence of these dietary components increases the likelihood of developing insulin resistance (Luan et al. 2020), acute cardiovascular conditions (Wang, Li, et al. 2024), gastrointestinal cancers (Dankner et al. 2024), and death (Carballo‐Casla et al. 2024; Chung et al. 2023). In contrast, foods abundant in nutrients such as fresh vegetables, fruits, and fermented dairy products could provide considerable health benefits. Enhancing the intake of these advantageous foods can prevent disorders related to glucose and lipid metabolism, decrease hepatic fat buildup and mitigate the “second hit” in NAFLD patients (Fridén et al. 2024; Keshavarz et al. 2022; Mucinski et al. 2024; Wang, Yan, et al. 2024). Specifically, coffee is known for its health‐promoting properties, exhibiting an inverse relationship with the severity of fibrosis in cases of NAFLD (Kositamongkol et al. 2021; Marventano et al. 2016; Morvaridi et al. 2020).

The gut microbiota exhibits a strong association with NAFLD. Dysbiosis within this microbiota may lead to increased absorption and subsequent release of proinflammatory substances. This process could catalyze the systemic translocation of endotoxins and elicit inflammatory responses in the liver (Chen and Vitetta 2020; Lai et al. 2025; Zaiss et al. 2021). Such imbalances also increase NAFLD risk by impairing the integrity of the intestinal mucosal barrier. When dysbiosis occurs, enhanced intestinal permeability facilitates the penetration of inflammatory factors into extraintestinal tissues, thereby promoting inflammation (Albhaisi et al. 2020; Amirkhizi et al. 2023; Kolodziejczyk et al. 2019; Talebi et al. 2023). Research by Loomba et al. (2017) noted marked variations in the prevalence of Firmicutes and Proteobacteria linked to liver diseases, with the former dominating in mild to moderate NAFLD stages and the latter in severe fibrotic stages. According to current works, dysbiosis is prevalent in NAFLD patients, and interventions such as probiotics or targeted therapies could restore microbial balance, alleviate inflammation, and fibrosis progression in NAFLD patients (Musazadeh et al. 2024; Song et al. 2024).

Dietary patterns critically shape the structure and functionality of gut microbiota, playing a crucial role in NAFLD prevention and management (Mousavi et al. 2022; Zheng et al. 2024). Diets rich in fiber promote beneficial bacteria such as Bifidobacterium while reducing potentially harmful taxa like *Streptococcus agalactiae* , *Mucispirillum schaedleri* , and *Alistipes indistinctus* , thereby alleviating NAFLD and hepatic inflammation (Chiou et al. 2022). Two key mechanisms mediate SCFA production and bile acid metabolism. First, fiber fermentation by Bifidobacterium and Lactobacillus generates SCFAs, particularly butyrate, which reduces pro‐inflammatory cytokines and enhances insulin sensitivity, thereby mitigating hepatic lipid accumulation (Li et al. 2021; Yang and Zhang 2021). Plant‐based foods (e.g., fruits, tea, coffee) and fermented products (e.g., Yogurt containing *Streptococcus thermophilus* and *bifidobacteria*) further reduce the Firmicutes/Bacteroidetes ratio, strengthening intestinal barrier integrity and improving hepatic lipid metabolism (Zhang et al. 2023). Second, diets high in red meat or fats disrupt bile acid metabolism by increasing secondary bile acids through dysbiosis, resulting in elevated Firmicutes/Proteobacteria ratios. This impairs farnesoid X receptor (FXR) and Takeda G‐protein‐coupled receptor 5 (TGR5) signaling, thereby increasing gut permeability, promoting endotoxin translocation, and exacerbating NAFLD via enhanced hepatic steatosis and fibrosis (Li et al. 2022; Yang and Zhang 2021). The involvement of SCFA‐ and bile acid‐related pathways, as modulated by dietary components within the DI‐GM framework, underscores the clinical utility of this index in tailoring targeted nutritional interventions for NAFLD.

The DI‐GM was developed by Kase et al. (2024) through a synthesis of 106 peer‐reviewed studies, which quantified the effects of dietary constituents on gut microbiota, including microbial diversity, abundance, and SCFA production. It is widely accepted as a robust tool for NAFLD research, providing a framework to assess gut‐liver axis and identify protective dietary strategies against NAFLD. Our study confirms a significant linear inverse connection of DI‐GM scores and NAFLD risk. Upon controlling for covariates in Model 3, individuals with greater DI‐GM scores demonstrated a diminished risk of NAFLD. Established dietary indices, including the HEI‐2015 and DASH, show inverse associations with NAFLD prevalence and liver fibrosis (Tian et al. 2022; Xiao et al. 2020; Zhang, Ding, et al. 2025). Our ROC curve analysis showed that the Dietary Index for Gut Microbiota (DI‐GM) achieved an AUC of 0.867 (95% CI: 0.857–0.877). This was significantly higher than the AUCs for HEI‐2015 (0.848) and DASH (0.847) (DeLong's test, both *p* < 0.0001). DI‐GM demonstrated superior predictive performance—exhibiting higher accuracy (0.772 vs. 0.746 for HEI‐2015 and 0.758 for DASH), as well as improved positive and negative predictive values. This enhanced precision may stem from its targeted emphasis on microbiota‐modulating foods, which influence NAFLD‐relevant pathways—such as SCFA production and gut barrier integrity—more directly than the broader, nutrient‐focused frameworks of HEI‐2015 or DASH. However, alternative explanations warrant consideration. For instance, the binary scoring architecture of the DI‐GM may be uniquely sensitive to polarized dietary patterns within our cohort. Furthermore, the possibility of residual confounding stemming from interrelated lifestyle behaviors cannot be entirely dismissed. These findings collectively highlight DI‐GM as a promising tool for evaluating dietary influences on NAFLD, especially for microbiota‐targeted interventions. To summarize, gut microbiota may influence NAFLD pathogenesis, which could be effectively predicted by DI‐GM.

Our findings were strengthened by a robust sample of 5283 individuals, which enhanced the credibility of the results. Subgroup analyses demonstrated insignificant interaction effects and consistent results with the main finding, suggesting uniform trends across various populations. Our research indicated that the DI‐GM consistently provided a protective effect against NAFLD, and dietary adjustments aimed at enhancing DI‐GM scores could potentially lower the risk of developing NAFLD. Extending beyond NAFLD, consistent patterns have been observed in related conditions, with DI‐GM showing inverse associations with liver disease (Zheng et al. 2025), constipation (Zhang, Bi, et al. 2025), depression (Zhang, Yang, et al. 2025), and biological aging (An et al. 2024). These findings reinforced the utility of DI‐GM in assessing the gut‐liver axis and identifying protective dietary strategies. Future studies should design targeted interventions based on DI‐GM to optimize gut microbiota composition and mitigate NAFLD progression.

This study demonstrated the clinical value of the association between DI‐GM and NAFLD risk, but several limitations exist. First, the absence of microbiome data in NHANES precluded direct validation of DI‐GM's mechanistic effects. Second, physical activity and genetic variations could modulate dietary responses, making it difficult to exclude reverse causality. For instance, individuals with diagnosed NAFLD might alter their diets to avoid harmful foods, potentially inflating the observed inverse association. While the exclusion of alternative liver conditions and adjustments for comorbidities mitigated some bias, prospective designs were essential to establish temporality. Third, the DI‐GM scores were obtained by 14 food intake variables obtained from mobile examination centers or 24‐h dietary recall interviews conducted via telephone. These methods are susceptible to recall bias, which may introduce selection bias. Fourth, DI‐GM may not account for all food components possibly linked to gut microbiota. Fifth, the scoring system used sex‐specific median values as the reference point, and subsequent research may investigate a more detailed stratification of these variables. Moreover, the U.S.‐centric NHANES population limited generalizability to other regions due to differences in dietary habits and environmental factors, potentially reducing the applicability of DI‐GM in global contexts. While this study revealed a notable inverse linear correlation between the DI‐GM and NAFLD, it did not definitively establish a causal link. Future research is warranted to corroborate our findings through more robust methodologies. Specifically, large‐scale prospective cohort studies and validations across heterogeneous regional populations are needed to ensure generalizability. Furthermore, randomized controlled trials are essential to evaluate the efficacy of DI‐GM‐guided dietary interventions in populations at high risk for NAFLD.

## 5 Conclusion

Consistent with our hypothesis, in this cross‐sectional analysis of the NHANES database, we identified a significant inverse linear relationship between elevated DI‐GM scores and NAFLD risk. The relationship persisted across subgroup analyses, thereby highlighting the potential protective effect of diets through gut microbiota. Our findings have significant implications for the prevention and clinical management of NAFLD, which require further validation in prospective studies and clinical trials. The DI‐GM, based on dietary components that modulate gut microbiota, provides a promising framework for designing nutritional interventions to prevent NAFLD. Specifically, encouraging the consumption of microbiota‐supportive foods, such as fiber‐rich vegetables, fermented dairy, and coffee, while limiting detrimental foods like red meat and high‐fat diets, could enable the integration of DI‐GM into clinical intervention, thereby enhancing gut‐liver axis health. In conclusion, these findings have demonstrated the possible role of DI‐GM in NAFLD prevention and management, which should be confirmed in future studies.

## Author Contributions

**Qingwan Yang:** conceptualization (lead), data curation (equal), formal analysis (equal), supervision (equal), writing – original draft (lead). **Xin Cai:** conceptualization (equal), methodology (lead), software (lead), writing – review and editing (equal). **Shanshan Li:** data curation (equal), formal analysis (equal), writing – review and editing (equal). **Zhenghua Xiao:** conceptualization (equal), funding acquisition (lead), project administration (lead), supervision (equal), writing – review and editing (equal).

## Funding

This study was supported by the National Natural Science Foundation of China (82460917), the Young Qihuang Scholars Program (National Administration of Traditional Chinese Medicine Education Development (2020) No. 7), the National Administration of Traditional Chinese Medicine's Project of High‐level Construction of Key TCM Disciplines (zyyzdxk‐2023187), the Construction of an Innovative Talent Team for Integrating Traditional Chinese and Western Medicine in the Prevention and Treatment of Digestive System Diseases at Guizhou University of TCM (Qian JiaoJi [2023] No. 017), and the Construction of an Innovative Talent Team for Integrating Traditional Chinese and Western Medicine in the Prevention and Treatment of Digestive System Diseases at Guizhou University of TCM (GUTCM TD Contract No. [2023] 001).

## Ethics Statement

The National Health and Nutrition Examination Survey (NHANES 1999–2018) was conducted under approval from the National Center for Health Statistics Research Ethics Review Board. As this secondary analysis exclusively utilized de‐identified NHANES data, no additional institutional review board approval was required for the present study.

## Consent

Written informed consent was obtained from all participants prior to data collection.

## Conflicts of Interest

The authors declare no conflicts of interest.

## Supporting information


**Tables S1–S3:** fsn371451‐sup‐0001‐TableS1‐S3.docx.

## Data Availability

The original data presented in the study are openly available at the National Health and Nutrition Examination Survey at https://wwwn.cdc.gov/nchs/nhanes/.
