# Supplementary material for: Association Between Nonalcoholic Fatty Liver Disease and the Dietary Index for Gut Microbiota: A Cross‐Sectional Study
Source: Food Sci Nutr. 2026 Jan 12;14(1):e71451. doi: 10.1002/fsn3.71451 (PMC12796509; doi:10.1002/fsn3.71451)
Supplement: Supplementary file 1 — Tables S1–S3: fsn371451‐sup‐0001‐TableS1‐S3.docx. [file FSN3-14-e71451-s001.docx]

**Table S1 Components of the DI-GM**

| Component | Included Foods within the Component | Scoring |
| --- | --- | --- |
| Beneficial to gut microbiota |  |  |
| Avocados | Avocados | For each component, a score of 1 if consumption at or above the sex-specific median, else 0 |
| Broccoli | Broccoli |  |
| Chickpea | Chickpeas |  |
| Coffee | Coffee |  |
| Cranberries | Cranberries |  |
| Fermented dairy | Yogurt, cheese, kefir, sour cream, buttermilk |  |
| Fiber | Not applicable |  |
| Green tea | Green tea |  |
| Soybean | Soy products—Soy milk, Tofu |  |
| Whole grains | Grains defined as whole grains, containing the entire grain kernel―the bran, germ, and endosperm |  |
| Unfavorable to gut microbiota |  |  |
| High-fat diet (% energy) | Not applicable | 0 if consumption at or above 40% energy from fat, else 1.  For each remaining component, a score of 0 if consumption at or above the sex-specific median, else 1 |
| Processed meat | Frankfurters, sausages, corned beef, and luncheon meat that are made from beef, pork, or poultry |  |
| Red meat | Beef, veal, pork, lamb, and game meat; excludes organ meat and cured meat |  |
| Refined grains | Refined grains that do not contain all of the components of the entire grain kernel |  |

**Table S2 Sensitivity Analysis of the Association Between DI-GM and NAFLD Risk Using Complete Cases (Excluding Samples with Missing Covariates)**

| Exposure | Model 1 | Model 2 | Model 3 |
| --- | --- | --- | --- |
|  | OR (95 % CI)  *P* value | OR (95 % CI)  *P* value | OR (95 % CI)  *P* value |
| DI-GM | 0.897 (0 864,0.931) <0.001 | 0.882(0.847,0.918) <0.001 | 0.926 (0.873,0.982) 0.010 |
| DI-GM quartile |  |  |  |
| Q1 | 1 | 1 | 1 |
| Q2 | 0.831(0.696.0.993) 0.041 | 0.873 (0.724,1.054) 0.157 | 0.973 (0.737,1.284) 0.845 |
| Q3 | 0.768 (0.642,0.917) 0.004 | 0.758(0.627,0.916) 0.004 | 0.925(0.701,1.220) 0.580 |
| Q4 | 0.599 (0.506,0.711) <0.001 | 0.572(0.476,0.687) <0.001 | 0.711 (0.544,0.931) 0.013 |
| P for trend | <0.001 | <0.001 | 0.010 |
| Model 1: Non-adjusted  Model 2: Adjusted for age, gender, ethnicity, educational level, marital status, poverty to income ratio  Model 3: Adjusted for age, physical activity MET, gender, ethnicity, educational level, marital status, poverty to income ratio, smoking status, body mass index, hypertension, diabetes, hyperlipidemia. | | | |

**Table S3 Sensitivity Analysis of the Association Between DI-GM and NAFLD Risk Using Multiple Imputation with Five Imputed Datasets and Pooled Results**

| Exposure | Model 1 | Model 2 | Model 3 |
| --- | --- | --- | --- |
|  | OR (95 % CI)  *P* value | OR (95 % CI)  *P* value | OR (95 % CI)  *P* value |
| DI-GM | 0.907(0.874,0.942) <0.001 | 0878(0.843,0.914) <0.001 | 0.930 (0.877,0.986) 0.015 |
| DI-GM quartile |  |  |  |
| Q1 | 1 | 1 | 1 |
| Q2 | 0.835 (0.700,0.997) 0.046 | 0.832 (0.690,1.003) 0.053 | 0.936 (0.712,1.229) 0.634 |
| Q3 | 0.815(0.682.0.973) 0.024 | 0.771 (0.639.0.931) 0.007 | 0.988 (0.751,1298) 0.928 |
| Q4 | 0.621 (0.524,0.737) <0.001 | 0.553 (0.461,0.665) <0.001 | 0.709(0.544,0.923) 0.011 |
| P for trend | <0.001 | <0.001 | 0.014 |
| Model 1: Non-adjusted  Model 2: Adjusted for age, gender, ethnicity, educational level, marital status, poverty to income ratio  Model 3: Adjusted for age, physical activity MET, gender, ethnicity, educational level, marital status, poverty to income ratio, smoking status, body mass index, hypertension, diabetes, hyperlipidemia. | | | |
